# Total 4EBP1 Is Elevated in Liver of Rats in Response to Low Sulfur Amino Acid Intake

**DOI:** 10.1155/2013/864757

**Published:** 2013-09-08

**Authors:** Angelos K. Sikalidis, Kevin M. Mazor, Minji Kang, Hongyun Liu, Martha H. Stipanuk

**Affiliations:** ^1^Division of Nutritional Sciences, Cornell University, Ithaca, NY 14853, USA; ^2^College of Animal Sciences, Zhejiang University, Hangzhou 310058, China

## Abstract

Translation initiation is known to be regulated by the binding of eukaryotic initiation factor 4E (eIF4E) by binding proteins (4EBPs), and there is evidence that amino acid deprivation and other cellular stresses upregulate 4EBP1 expression. To pursue the question of whether diets limited in an essential amino acid lead to induction of 4EBP1 expression in vivo, diets that varied in methionine and cystine content were fed to rats for 7 days, and 4EBP1 mRNA and protein levels and 4EBP1 phosphorylation state were determined. Total 4EBP1 mRNA and protein abundance increased in liver of rats with severely deficient intakes of sulfur amino acids (0.23% or 0.11% methionine without cystine) but not in animals with a less restricted intake of sulfur amino acids (0.11% methionine plus 0.35% cystine) but a similarly restricted intake of total diet (53 to 62% of control). The amount of 4EBP1 binding activity (**α** + **β** forms) was elevated in liver of rats fed sulfur amino acid-deficient diets, whereas the hyperphosphorylation of 4EBP1 was not affected by dietary treatment. Results suggest that changes in total 4EBP1 expression should be considered when examining mechanisms that attenuate protein synthesis during amino acid deficiency states.

## 1. Introduction

Regulation of protein synthesis in eukaryotic cells occurs primarily at the initiation step of mRNA translation, during which ribosomal subunits are recruited to the mRNA through the action of eukaryotic initiation factors (eIFs). The regulation of mRNA translation initiation plays critical roles in the regulation of cell division, differentiation, growth, survival, and apoptosis, and dysregulation of translation initiation is associated with cancer, obesity, insulin resistance, and impaired responses to various stress situations [1–7]. The two best understood mechanisms for regulation of mRNA translation initiation are the regulation of formation of the ternary complex, which consists of the methionine-charged initiator tRNA, eIF2, and GTP, and the regulation of assembly of the eIF4F complex, which involves the association of the mRNA 5′ cap-binding protein eIF4E with eIF4G and eIF4A.

Phosphorylation of the alpha subunit of eIF2 (eIF2*α*) blocks ternary complex formation, thereby blocking formation of the 43S preinitiation complex and suppressing global translation. Mammals have four eIF2*α* kinases that are activated by different types of cellular stress, including the unfolded protein response and amino acid deprivation, such that downstream effects of eIF2*α* phosphorylation are shared among different stress-response pathways [8]. The global attenuation of translation that results from a lack of 43S preinitiation complex paradoxically increases the translation of a subset of mRNAs, including that encoding activating transcription factor 4 (ATF4) [9]. Upregulation of the translation of ATF4 and other target proteins can then lead to increased transcription of stress-related genes (such as *Atf3, Asns, Cebpb*, and *Trib3*), allowing the cell to synthesize the subset of proteins needed to respond to the stress that initiated the response [10–12].

Formation of the eIF4F complex is regulated by competition between eIF4E binding proteins (4EBPs) and eIF4G for binding to eIF4E. The 4EBPs compete with eIF4G for a shared binding site of eIF4E, such that the binding of 4EBPs and eIF4G to eIF4E is mutually exclusive. In response to the presence of growth factors and nutrients, mechanistic target of rapamycin complex 1 (mTORC1) phosphorylates 4EBP, which leads to further phosphorylation of additional residues of 4EBP [13]. The hyperphosphorylation of 4EBP dramatically reduces its affinity for eIF4E and thereby promotes its association with eIF4G to form the translationally competent eIF4F complex, which recruits the 43S preinitiation complex to the mRNA. 4EBP hyperphosphorylation diminishes the capacity of eIF4E to bind TOP-like mRNAs much more than other mRNAs, which explains why mTORC1 inhibition results in a marked inhibition of translation of a subset of mRNAs that tend to encode proteins associated with translation (e.g., cytoplasmic ribosomal proteins) and a more modest suppression of the translation of other mRNAs [14].

In mammals, the 4EBP family consists of 3 proteins, 4EBP1, 4EBP2, and 4EBP3. The best characterized 4EBP is 4EBP1, which contains six known Ser/Thr phosphorylation sites, two of which are phosphorylated directly by mTORC1 [13, 15, 16]. 4EBP1 is most abundant in tissues involved in glucose and lipid homeostasis, including adipose tissue, pancreas, muscle, and liver [17], whereas 4EBP2 is expressed ubiquitously [18]. In contrast to 4EBP1 and 4EBP2, 4EBP3 lacks a conserved N-terminal regulatory motif (RAIP) that is critical for phosphorylation of 4EBP1/2 at the mTORC1-regulated sites [13, 19] and may play a different cellular role than 4EBP1 and 4EBP2 [20].

The regulation of 4EBP1 expression in various tissues has not been studied extensively although studies with cell lines have demonstrated TGF*β*-SMAD4-mediated [21], MYC-mediated [22], eIF2*α* kinase-dependent [23], or ATF4-dependent [6] upregulation of *4Ebp1* gene transcription. In addition, *4Ebp1* gene transcription was negatively regulated by induction of Egr1 expression in various cells in response to activation of ERK/mitogen-activated protein kinase or PI3K signaling pathways [24, 25]. Despite the complexity of the body's responses to various stress situations, the GCN2 (general control nonderepressible 2, also known as eIF2*α* kinase 4) ATF4 pathway is well known to regulate many responses to amino acid deprivation [26–28] and is a likely mediator of the induction of 4EBP1 by sulfur amino acid deprivation.

The *4Ebp1* gene contains two CCAAT-enhancer binding protein-activating transcription factor (C/EBP-ATF) response elements (CARE), and the upregulation of 4EBP1 mRNA [23] or protein [26] abundance in response to amino acid deficiency has been reported for cells cultured in medium deficient in an essential amino acid. Upregulation of 4EBP1 has also been shown to occur in murine tissues and in MIN6 cells following induction of endoplasmic reticulum (ER) stress with thapsigargin or tunicamycin [6]. Expression of a dominant-negative form of ATF4 in MIN6 cells suppressed 4EBP1 induction by thapsigargin, whereas expression of wild-type ATF4 dramatically induced 4EBP1 expression, supporting a critical role for ATF4 in induction of 4EBP1 expression [6]. Furthermore, 4EBP1 mRNA levels were not increased by thapsigargin in *Atf4−/−* murine embryonic fibroblasts; the transcriptional inhibitor actinomycin D completely blocked induction of 4EBP1 protein by thapsigargin in MIN6 cells; disruption of both ATF4 binding sites in the *4Ebp1* promoter abolished reporter gene expression in MIN6 cells [6].

Thus, it seems likely that stress conditions, such as amino acid deprivation, may affect translational control by increased 4EBP1 abundance, which could inhibit eIF4F assembly, as well as by the well-established mechanism that involves diminished formation of ternary complex, with both the decrease in ternary complex formation and the increase in 4EBP1 abundance dependent on the eIF2*α*/ATF4 signaling pathway. Because we previously saw a marked upregulation of eIF2*α* phosphorylation and induction of stress responses in liver of rats fed a sulfur amino acid-deficient soy protein diet [27], we decided to explore the expression of hepatic 4EBP1 and its association with eIF2*α* phosphorylation and mTOR-mediated 4EBP1 hyperphosphorylation/inactivation in rats fed amino acid-based diets that differed in methionine and cystine content.

## 2. Materials and Methods

### 2.1. Animals and Diets

The study was conducted using male Sprague-Dawley rats that weighed approximately 110 g (~5 weeks of age) and were purchased from Harlan Sprague Dawley (Indianapolis, IN, USA). Animal procedures were approved by the Cornell University Institutional Animal Care and Use Committee. Rats were housed individually in polycarbonate cages containing corncob bedding in a holding room maintained at 20°C and 60–70% humidity. The room was lighted from 21:00 to 09:00 h.

Rats were fed diets that contained crystalline amino acids instead of protein. The compositions of the experimental diets, which varied in methionine and cystine levels, are shown in Table 1. Diets were prepared by Dyets Inc. (Bethlehem, PA, USA). Sulfur amino acid levels were based on previous rat studies that demonstrated that 2.3 g methionine is adequate to meet the absolute requirement of growing rats for methionine (i.e., the requirement for methionine when cyst(e)ine is not limited in the diet) [29, 30]. For diet preparation, the powdered diets were mixed with an equal volume (1 L/kg diet) of hot agar solution (3 g/L), and the mixture was cooled at room temperature, refrigerated, and cut into cubes for feeding. All rats were fed a complete amino acid-based diet (0.23% M/0.35% C) for 1 week for acclimation purposes prior to experimental group assignment. At the end of the adaptation week, rats were randomly assigned to four experimental groups, which were fed the complete amino acid-based diet (0.23% M/0.35% C), an amino acid-based diet that contained 0.11% l-methionine and 0.35% l-cystine (0.11% M/0.35% C), a diet that contained 0.23% l-methionine but no cystine (0.23% M), or a diet that contained 0.11% l-methionine but no cystine (0.11% M). Experimental diets were fed for one week, with fresh diet being given at the beginning of the dark cycle each day. Feed intake and body weights were measured daily.

At the end of the dietary treatment period (i.e., between 13:00 and 14:00 h on day 8), rats were anesthetized with CO_2_, and liver was rapidly removed, rinsed with ice-cold saline, and frozen in liquid nitrogen. Frozen tissue was stored at −80°C until analyses were performed.

### 2.2. Measurement of Hepatic Nonprotein Bound Thiol Levels

Nonprotein-bound intracellular cyst(e)ine levels were measured by the modified acid ninhydrin method of Gaitonde [31] as described by Dominy et al. [32] after reduction of disulfides with dithiothreitol. Nonprotein-bound intracellular glutathione levels were measured by the HPLC method of Cereser et al. [33] after reduction of tissue acid supernatants with NaBH_4_.

### 2.3. Analysis of 4EBP1, rpS6, and eIF2*α* Protein Levels and Phosphorylation State and eIF4E Protein Level

Rat liver samples were homogenized in TNES buffer with mammalian protease inhibitor cocktail (Sigma), PhosStop phosphatase inhibitor cocktail (Roche Applied Sciences), and 50 mM NaF to give a 20% (w/v) homogenate. Homogenates were centrifuged at 18,000 ×g for 20 min at 4°C, and the protein concentration of the soluble fraction was determined using the BCA protein assay (Thermo Scientific). Fifty micrograms of protein per lane was resolved by SDS-PAGE and transferred onto a PVDF membrane (Millipore Corp.). Membranes were immunoblotted using antibodies to 4EBP1, ribosomal protein S6 (rpS6), rpS6-P (Ser^240/244^), eIF2*α*, eIF2*α*-P (Ser^51^), eIF4E, and actin (all from Cell Signaling, Danvers, MA, USA). Visualization of bands was accomplished either using horseradish peroxidase-coupled secondary antibodies (1 : 25,000 dilution in 5% (w/v) dry fat-free milk in 1× TBST) and chemiluminescent substrates (West Dura, Pierce) with exposure to autoradiography film or using IRDye-labeled secondary antibody (1 : 15,000) in Odyssey blocking buffer plus 0.1% Tween-20 and 0.01% SDS (Li-Cor Biosciences, Lincoln, NE, USA). Film images were digitized and analyzed using NIH Image 1.63 software, and Odyssey images were analyzed using Li-Cor Odyssey V3.0 software. Band intensities were normalized against corresponding bands for *β*-actin.

### 2.4. Analysis of 4EBP1 and eIF4E mRNA Levels

RNA was isolated using the RNeasy Mini Kit according to the manufacturer's directions (Qiagen). Complementary DNA was reverse transcribed using Applied Biosystems High Capacity cDNA kit (Applied Biosystems) and quantified using Power Sybr Green (Applied Biosystems) in conjunction with a Roche 480 Lightcycler (Roche Diagnostics). Primers for 4EBP1 mRNA were forward (5′-3′) GATGAGCCTCCCATGCAG and reverse (5′-3′) CCATCTCAAACTGTGACTCTTCA. Primers for eIF4E mRNA were forward (5′-3′) GCAATATGGACGACTGAATGTG and reverse (5′-3′) GTGTCTGCGTGG GACTGATA. Values for mRNA were normalized to values for tubulin.

### 2.5. Statistical Analysis

Results were analyzed by one-way ANOVA, and post hoc tests were done by Tukey's procedure. Due to unequal variance, data for rpS6 and 4EBP1 were transformed to square roots prior to statistical analysis. Differences were accepted as significantly different at *P* ≤ 0.05.

## 3. Results

### 3.1. Body Weight and Feed Intake

**Figure 1:**
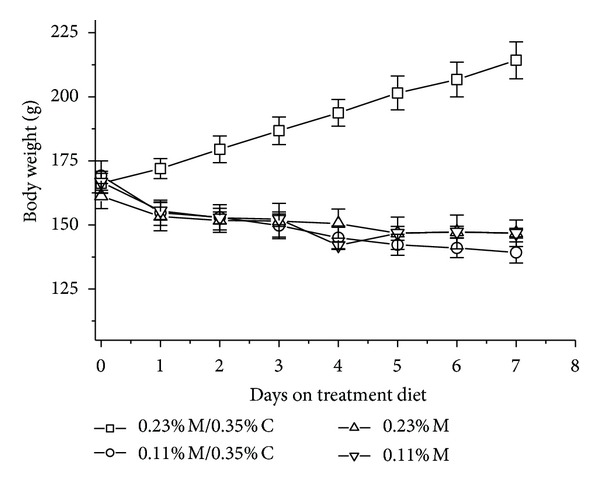
Effects of feeding diets differing in sulfur amino acid levels on body weight of rats. Values are means ± SEM for 4 rats.

Rats fed any of the three amino acid deficient diets (0.11% M/0.35% C, 0.23% M, 0.11% M) exhibited lower feed intake (Table 2) as well as lower body weight (Figure 1) compared to control rats fed the complete 0.23% M/0.35% C diet. Rats fed diets containing 0.11% M/0.35% C, 0.23% M, and 0.11% M consumed 60%, 53%, and 62%, respectively, as much diet as control rats. Notably, feed intake of these three sulfur amino acid deficient groups was similar despite the differences in dietary sulfur amino acid deficiency. Rats fed the sulfur amino acid deficient diets lost weight over the 7-day feeding period, whereas rats fed the complete amino acid diet gained an average of 6.9 ± 0.2 g per day. Weight loss for the various sulfur amino acid deficient groups paralleled the magnitude of the sulfur amino acid deficiency (Table 2).

### 3.2. Liver Weights

In absolute weight, liver was 67%, 57%, and 51% of control for the 0.11% M/0.35% M, 0.23% M, and 0.11% M groups, respectively (data not shown). When adjusted for body weight, the liver weights were not significantly different (*P* > 0.05) among groups although they still tended to decrease with the degree of sulfur amino acid deficiency (94%, 84%, and 78% of control for the 0.11% M/0.35% M, 0.23% M, and 0.11% M groups, resp.).

### 3.3. Hepatic Cysteine and Glutathione

**Figure 2:**
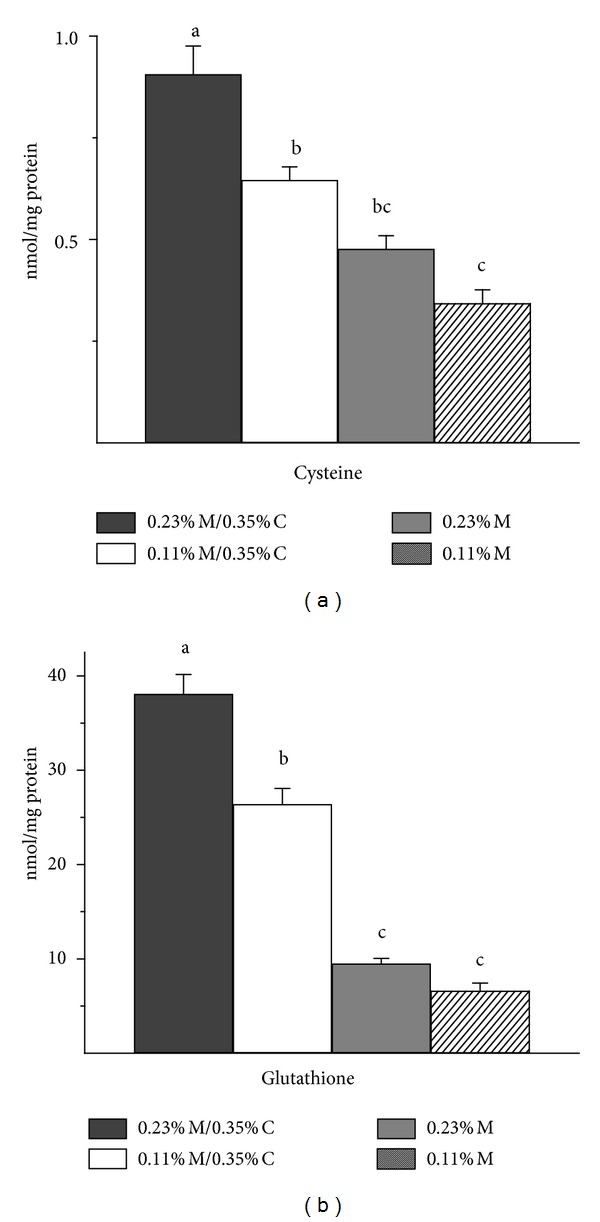
Effects of feeding diets differing in sulfur amino acid levels on hepatic nonprotein-bound cysteine and glutathione levels. Values are means ± SEM for 4 rats. Values represented by bars not labeled with the same letter are significantly different at *P* ≤ 0.05 by ANOVA and Tukey's comparison.

As shown in Figure 2, hepatic thiol levels were markedly lower for rats fed the amino acid deficient diets than for rats fed the control diet. Hepatic total cysteine levels decreased in a stepwise fashion with each decrease in sulfur amino acid content of the diet, with rats fed the 0.11% M/0.35% C diet having 71%, rats fed the 0.23% M diet having 52%, and rats fed the 0.11% M diet having 38% of control cysteine level. Changes in hepatic total glutathione levels tended to parallel total cysteine levels, being 69%, 25%, and 18% of control, respectively, for rats fed the 0.11% M/0.35% C, 0.23% M, and 0.11% M diets.

### 3.4. Expression of Hepatic 4EBP1 and eIF4E

**Figure 3:**
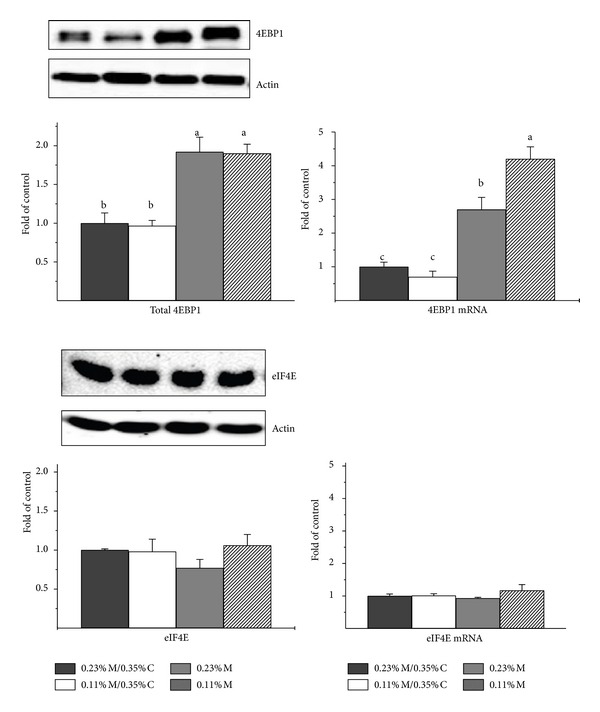
Effects of feeding diets differing in sulfur amino acid levels on the abundance of 4EBP1 and eIF4E protein and mRNA in liver of rats. Values are means ± SEM for 4 rats. Values represented by bars not labeled with the same letter are significantly different at *P* ≤ 0.05 by ANOVA and Tukey's comparison. Representative western blots are shown; these were run by loading equal amounts of total soluble protein per lane with the order of samples being the same as for the bar graphs. Protein values were normalized by *β*-actin, whereas mRNA abundances were normalized to tubulin.

Compared to control rats, both hepatic 4EBP1 mRNA abundance and hepatic total 4EBP1 protein abundance were 2- to 4-fold higher in rats fed the two most deficient diets (0.23% M and 0.11% M) (Figure 3). On the other hand, the abundances of total 4EBP1 protein and of 4EBP1 mRNA were not different from control in liver of rats fed the 0.11% M/0.35% C diet. Because an increase in 4EBP1 may have little effect on translation initiation if the amount of eIF4E changes in a similar direction, we also measured the abundance of eIF4E. However, neither eIF4E protein abundance nor mRNA abundance was affected by dietary treatment (Figure 3).

### 3.5. Phosphorylation State of 4EBP1

**Figure 4:**
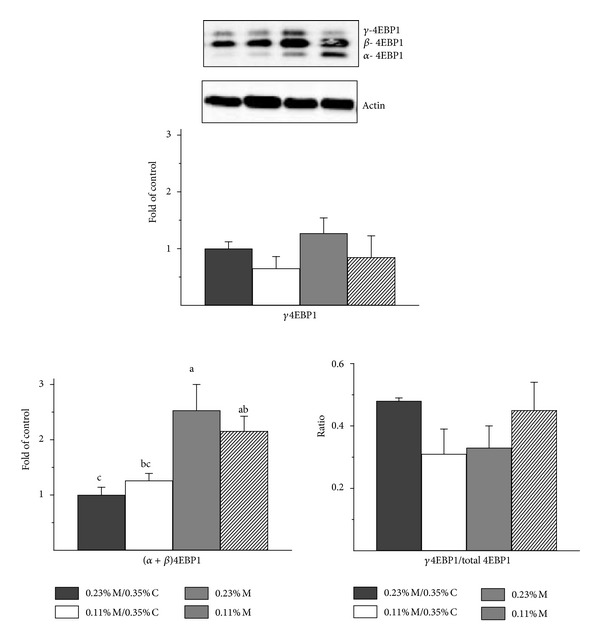
Effects of feeding diets differing in sulfur amino acid levels on the 4EBP1 phosphorylation status in liver of rats. Values are means ± SEM for 4 rats. Values represented by bars not labeled with the same letter are significantly different at *P* ≤ 0.05 by ANOVA and Tukey's comparison; values for *γ*4EBP1/total 4EBP1 were transformed to square roots prior to statistical analysis. A representative western blot is shown; equal amounts of total soluble protein were loaded per lane with the order of samples, being the same as for the bar graphs. A higher percentage of polyacrylamide and a longer run time were used for electrophoresis to obtain better separation of the bands than for the western blots shown in Figure 3. The amounts of *α*4EBP1, *β*4EBP1 and *γ*4EBP1 were normalized by actin. The ratio of *γ*4EBP1 to total 4EBP1 is the ratio of the density of the *γ*4EBP1 band to the sum of the densities for all three 4EBP1 bands.

The active, hypophosphorylated (alpha and beta) forms of 4EBP1 were higher in liver of rats fed the two most deficient diets than in liver of control rats, following a trend similar to that for total 4EBP1 abundance (Figure 4). Neither the abundance of the hyperphosphorylated gamma form of 4EBP1 nor the ratio of gamma 4EBP1 to total 4EBP1 differed among the four groups (*P* > 0.05).

### 3.6. mTORC1 and eIF2*α* Kinase Activation

**Figure 5:**
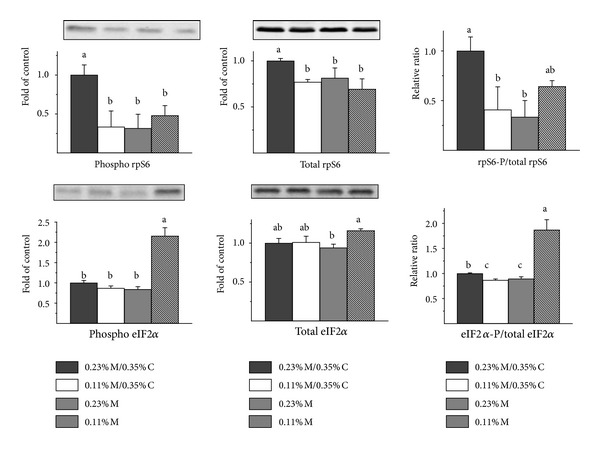
Effects of feeding diets differing in sulfur amino acid levels on rpS6 phosphorylation status and eIF1*α* phosphorylation status in liver of rats. Values are means ± SEM for 4 rats. Values represented by bars not labeled with the same letter are significantly different at *P* ≤ 0.05 by ANOVA and Tukey's comparison; values for phospho-eIF2*α* and eIF2*α*P/total eIF2*α* were transformed to square roots prior to statistical analysis. Representative western blots are shown; equal amounts of total soluble protein were loaded per lane with the order of samples the same as for the bar graphs. The amounts of the indicated proteins were normalized by actin. To avoid the dependence of ratios on exposure times with the different antibodies, rpS6-P/total rpS6 ratios and eIF2*α*-P/total eIF2*α* ratios were calculated as relative ratios after first expressing densities as fold of the control group.

Although the failure to observe a difference in the proportion of total 4EBP1 in the hyperphosphorylated form in rats fed adequate and deficient diets suggests that there was no change in mTORC1 activity, it is possible that the ratio of *γ*-4EBP1 to total 4EBP1 was impacted by the doubling of total 4EBP1 levels in rats fed the diets with 0.11% M or 0.23% M without cysteine. To further assess mTORC1 activation, we examined the phosphorylation state of the rpS6 (ratio of rpS6-P to total rpS6) as another indicator of mTORC1 activation state (Figure 5). The p70S6 kinase (S6K) is a direct substrate of mTORC1, and rpS6 is the target of p70S6 kinase. The ratio of rpS6-P to total rpS6 was significantly lower than control (*P* < 0.05) in liver of rats fed the 0.11% M/0.35% C and 0.23% M diets but not in liver of rats fed the 0.11% M diet, perhaps due to the concurrent decrease in total rpS6 in rats fed the latter diet. The amounts of rpS6-P and total rpS6 were significantly lower in liver of rats fed all three of the amino acid deficient diets. Although the ratio of rpS6-P to total rpS6 was not significantly lower in the 0.11% M group than in the control group, it was similar to the ratios for the 0.11% M/0.35% C and 0.23% M diet groups.

Because amino acids can also signal via activation of GCN2 and because eIF2*α* kinase activation may be required for an increase in 4EBP1 expression, we also measured eIF2*α* phosphorylation. An increase in eIF2*α* phosphorylation was observed only in liver of rats fed the 0.11% M diet. Surprisingly, rats fed the other amino acid deficient diets had hepatic eIF2*α*-P to total eIF2*α* ratios that were similar to or less than those of control rats.

## 4. Discussion

Rats given the control diet gained an average of almost 7 g per day, but those given the sulfur amino acid deficient diets did not even maintain their starting body weights. Weight loss was partially due to reduced feed intake, as established by a follow-up comparison of rats fed the 0.11% M diet ad libitum with a group pair-fed the control diet for 7 days. In the follow-up study (data not reported), pair-fed rats gained 1.3 ± 0.2 g per day, whereas rats given free access to the 0.11% M diet lost 4.6 ± 0.3 g per day (comparable to the 4.3 ± 0.9 g per day lost by the 0.11% M group in the main study). Feed intake was similar in the three sulfur amino acid deficient groups, so the contribution of reduced feed intake was presumably similar for all three groups. Lack of adequate sulfur amino acids, however, contributed to weight loss in a stepwise manner, with weight loss being greatest in those with the lowest intakes.

The inadequacy of dietary sulfur amino acids was also reflected in the reduced hepatic cysteine and glutathione levels, which also exhibited a stepwise decrease that followed the magnitude of the deficit. The effects of dietary sulfur amino acid level on growth of young rats is consistent with earlier studies demonstrating that a diet must provide approximately 5 g methionine equivalents per kg diet, with at least half of this present as methionine, in order to support adequate sulfur amino acid status and maximal growth of Sprague Dawley rats during their 6th to 8th weeks [27, 29, 30]. The control diet essentially met this requirement, but the other diets were low either in total sulfur amino acids or in methionine.

Total hepatic 4EBP1 mRNA and 4EBP1 protein abundances were both elevated in liver of rats fed the 0.23% M or 0.11% M diet but not in rats fed the 0.11% M/0.35% C diet, despite all three groups having similarly reduced feed intakes compared to the control group. This suggests that essential amino acid deficiency, not just low feed intake, is necessary for induction of 4EBP1 expression in liver of intact rats. Other observations in our laboratory strengthen this conclusion. Rats fed a protein-free diet had nearly 60% higher (*P* ≤ 0.05) hepatic 4EBP1 levels as well as a 200% higher (*P* ≤ 0.05) level of 4EBP1 mRNA than rats fed a control 20% soy protein diet. Rats deprived of food for 60 h, on the other hand, exhibited low levels of 4EBP1 mRNA (45% of control, *P* ≤ 0.05) and of 4EBP1 protein (39% of control, *P* ≤ 0.05) (Sikalidis, Mazor and Stipanuk, unreported observations). Furthermore, additional analyses of liver of rats fed a 10% soy protein with 0.34% l-methionine (control) or without supplemental methionine (deficient) [27] demonstrated a robust induction of hepatic 4EBP1 mRNA and 4EBP1 protein levels in the sulfur amino acid-deficient group fed the unsupplemented diet (to 3.4- and 2.4-times control levels, resp.); in this study the sulfur amino acid-deficient rats consumed 83% as much feed and gained 35% as much weight as did the control rats over the 7-day treatment period. Thus, the upregulation of 4EBP1 expression in liver of rats appears to occur in response to mild as well as severe deficiencies of dietary sulfur amino acids as long as the rats are consuming at least half as much feed as control rats (Sikalidis and Stipanuk, unreported data). In the case of starvation or lack of a source of exogenous energy, it is likely that amino acid concentrations in liver increase due to increased breakdown of muscle protein, which is consistent with our observation of higher cysteine and glutathione levels in liver of starved rats than control rats. Interestingly, we did not see changes in total 4EBP1 in gastrocnemius muscle of rats fed a protein-free diet or in those deprived of feed although both showed a dramatic decrease in 4EBP1 hyperphosphorylation. This suggests that the regulation of *4Ebp1* gene expression may occur in a tissue-specific manner.

Severe deficiencies of sulfur amino acids (i.e., intakes ≤ 0.023 g Met equivalents per day) were always associated with elevated hepatic levels of both total and hypophosphorylated 4EBP1, except in the case of starvation. The degree of sulfur amino acid deficiency relative to total feed (or energy) deficiency seems to play a role; however, modest deficiencies appeared to have a greater effect when total feed (energy) intake was more adequate (i.e., rats fed a 10% soy protein diet in our previous study with feed intake equivalent to 83% of control and Met equivalent intake of 0.043 g/day exhibited elevated hepatic 4EBP1, whereas rats fed the 0.11% M/0.35% C diet with feed intake equivalent to 60% of control and Met equivalent intake of 0.063 Met equivalents per day did not exhibit a change in hepatic 4EBP1 expression). The apparent interaction between sulfur amino acid intake and total feed (energy) intake might be explained by greater muscle proteolysis in the rats with the lower feed intakes. However, additional studies will be needed to confirm this hypothesis.

It is reasonable to conclude that the increase in 4EBP1 observed in liver of rats fed sulfur amino acid deficient diets would contribute to the suppression of cap-dependent mRNA translation/protein synthesis. The abundances of eIF4E mRNA and eIF4E protein in liver of rats fed the sulfur amino acid deficient diets were not different from those in liver of control animals, indicating that there was no compensatory increase in eIF4E expression to offset the increase in 4EBP1 expression in rats fed diets severely restricted in sulfur amino acids. Assessment of changes in the abundance of the hypophosphorylated forms of 4EBP1, the forms that are active in binding eIF4E, indicated that higher levels of total 4EBP1 expression were associated with higher abundance of the alpha plus beta forms of 4EBP1. Thus, these results suggest that diets limited in essential amino acids (at least, sulfur amino acids) may elevate amounts of total 4EBP1, which could in turn reduce cap-dependent protein synthesis. Although we did not measure the association of eIF4E with either 4EBP1 or eIF4G in this study, numerous studies with animals have shown that an increase in the abundance of the active forms of 4EBP1 is accompanied by increased association of eIF4E with 4EBP1 and decreased association of eIF4E with eIF4G to form the translationally active eIF4F complex [34–38].

In this study, the hyperphosphorylation of 4EBP1, which is dependent upon mTORC1, was not affected by the different diets. Neither the relative abundance of gamma 4EBP1 nor the ratio of gamma 4EBP1 to total 4EBP1 was different among the four diet groups. This seems to indicate that mTORC1 activity was not suppressed in liver of rats fed the sulfur amino acid deficient diets, despite their lower feed (energy) intake and lower essential amino acid intake. Using phosphorylation of rpS6 as another measure of mTORC1 activity state, the ratio of phosphorylated rpS6 to total rpS6 was lower in rats fed the sulfur amino acid deficient diets, suggesting that mTORC1 activity was reduced in rats with lower energy and sulfur amino acid intakes. Other reports have also shown that the magnitude of increased phosphorylation of rpS6 was much higher than that of 4EBP1 in response to hepatic mTORC1 activation such that it may be more difficult to observe a decrease in 4EBP1 phosphorylation, leading to less gamma 4EBP1, than to observe a decrease in rpS6 phosphorylation by rpS6 kinase [39, 40]. Nevertheless, the overall outcome was clearly a greater abundance of the hypophosphorylated forms of 4EBP1 (i.e., alpha + beta 4EBP1) in liver of rats fed diets severely limiting in sulfur amino acids.

The upregulation of 4EBP1 protein was tightly associated with upregulation of 4EBP1 mRNA abundance. Because cell culture studies have suggested that 4EBP1 expression may be upregulated by a GCN2-eIF2*α*-ATF4 mechanism [23, 26] and because the role of ATF4 in *4Ebp1* gene transcription has been clearly established in MIN6 cells [6], we assessed the phosphorylation of eIF2*α*. In contrast to the close associations observed earlier between eIF2*α* phosphorylation and levels of ATF4, ATF3, ASNS, SLC7A11, CARS, and CTH protein abundance in liver of rats fed diets limited in sulfur amino acids [27], we did not observe a consistent association of eIF2*α* phosphorylation with increased 4EBP1 protein levels. Although both eIF2*α* phosphorylation and total 4EBP1 expression were upregulated in liver of rats fed the 0.11% M diet compared to the 0.23% M/0.35% C diet, 4EBP1 expression but not eIF2*α* phosphorylation was upregulated in liver of rats fed the 0.23% M diet. These results might be explained by differences in the time courses of eIF2*α* phosphorylation in liver of rats fed the two diets; rats fed the 0.23% M diet may have been able to adapt more adequately such that eIF2*α* phosphorylation was reduced by 7 days. Other investigators have reported that eIF2*α* phosphorylation and increases in ATF4 abundance tend to peak prior to peak expression of the downstream stress response proteins [6, 41–43]. Another possibility is that a pathway not involving eIF2*α* phosphorylation can induce *4Ebp1* gene expression. In this regard, methionyl-tRNA synthetase, which adds methionine to both initiator and elongator tRNA^Met^, has been shown to be a substrate for GCN2, at least under some conditions, and phosphorylation of methionyl-tRNA synthetase by GCN2 prevents binding of tRNA to the enzyme, reducing the aminoacylation of both initiator and elongator tRNA^Met^ [44, 45]. Further work will be required to elucidate whether increases in hepatic total 4EBP1 expression in response to sulfur amino acid-limited diets is independent, or partially independent, of eIF2*α* phosphorylation status. Interestingly, GCN2 phosphorylation of either eIF2*α* or methionyl-tRNA synthetase would negatively impact ternary/preinitiation complex formation.

Whether or not 4EBP1 expression would be increased in response to deficiencies of essential amino acids other than sulfur amino acids was not addressed in this work, but studies performed in cell culture systems suggest that this would be the case [26]. Deficiencies of any essential amino acid are known to activate GCN2's kinase activity although responses to particular amino acids tend to vary with cell type [11, 26, 46, 47]. Using HepG2 cells, Palii et al. [26] showed that removal of any single essential amino acid from the medium resulted in an increase in total 4EBP1 abundance but variable increases in eIF2*α* phosphorylation or ATF4 abundance, suggesting likely crosstalk among signaling pathways that are not all impacted similarly by lack of a particular essential amino acid. Even eIF2*α* phosphorylation and ATF4 abundance were poorly correlated, with leucine and threonine deficiency yielding the higher degrees of eIF2*α* phosphorylation but valine and methionine yielding the highest levels of ATF4 abundance [26]. It is possible that a methionine deficiency could have some unique effect beyond those mediated by GCN2, perhaps via reduced availability of charged initiator tRNA^Met^ as discussed above.

## 5. Conclusions

Results reported here for hepatic 4EBP1 expression in rats fed sulfur amino acid deficient diets further support a physiological role for changes in 4EBP1 expression in the response of animals to a deficiency of essential amino acids. In both this study and the previous study in intact rats, higher total 4EBP1 abundance was not associated with an increase in the proportion of the 4EBP1 in the hyperphosphorylated, inactive form. Thus, changes in hepatic 4EBP1 expression led to parallel changes in the abundance of the active, hypophosphorylated forms of 4EBP1 available to bind eIF4E and block cap-dependent translation initiation. It seems likely that elevated levels of 4EBP1 are involved in regulation of hepatic protein synthesis under conditions of severe limitations of one or more essential amino acids in the face of only modest limitations of energy and other nutrients.

Induction of 4EBP1 expression in liver of rats fed diets severely limited in sulfur amino acids did not appear to require sustained eIF2*α* phosphorylation, although we cannot rule out a contribution of eIF2*α* phosphorylation to 4EBP1 induction. Despite similar levels of total 4EBP1 in liver of rats fed the 0.23% M and 0.11% M diets, the abundance of 4EBP1 mRNA was higher in liver of rats fed the 0.11% M diet, which also had elevated levels of phosphorylated eIF2*α*, than in liver of rats fed the 0.23% M diet, which did not have elevated levels of phosphorylated eIF2*α*. We are currently undertaking studies in cell culture models to address how a sulfur amino acid deficiency is sensed and what signaling pathways are involved in increased expression of 4EBP1. Regardless of the underlying amino acid sensing and signaling mechanisms, it is nevertheless clear from this study that changes in total 4EBP1 expression should be considered when examining mechanisms that attenuate protein synthesis during amino acid deficiency states.

## Figures and Tables

**Table 1:** Composition of experimental diets.

	Control			
	0.23% M/0.35% C*	0.11% M/0.35% C	0.23% M	0.11% M
Ingredient	g/kg diet			
L-Amino acid mix^†^	172	172	172	172
L-Methionine	2.3	1.1	2.3	1.1
L-Cystine	3.5	3.5	0	0
Cornstarch	389.9	389.9	389.9	389.9
Dextrinized corn starch	155	155	155	155
Sucrose	102.4	103.6	105.9	107.1
Cellulose	50	50	50	50
Soybean oil	70	70	70	70
* tert-*Butylhydroquinone	0.01	0.01	0.01	0.01
Mineral mix	35	35	35	35
Vitamin mix	10	10	10	10
Choline bitartrate	2.5	2.5	2.5	2.5
Sodium bicarbonate	7.4	7.4	7.4	7.4
Methionine equivalents, g/kg^‡^	6.6	5.4	2.3	1.1

*M: L-methionine, C: L-cystine.

^†^L-amino acid mix (g/kg): L-arginine 6.3, L-histidine 4.5, L-tyrosine 9.2, L-phenylalanine 8.7, L-leucine 15.3, L-Isoleucine 8.4, L-valine 9.9, glycine 3.1, L-proline 20.4, L-glutamic acid 36.2, L-alanine 4.5, L-aspartic acid 11.3, L-serine 9.4, L-lysine-HCl 16.1, L-threonine 6.6, and L-tryptophan 2.1. Sodium bicarbonate was added to neutralize lysine-HCl.

^‡^Methionine equivalents = g L-methionine + (g L-cyst(e)ine × 149/120).

**Table 2:** Weight change and feed intake of rats fed diets differing in sulfur amino acid content.

	Dietary group			
	0.23% M/0.35% C Control	0.11% M/0.35% C	0.23% M	0.11% M
Mean weight change (g/d)	6.9 ± 0.2^a^	−1.1 ± 0.7^b^	−2.9 ± 1.1^bc^	−4.3 ± 0.9^c^
Mean feed intake (g/d)	19.3 ± 0.5^a^	11.6 ± 0.6^b^	10.2 ± 1.2^b^	11.9 ± 0.7^b^
Feed intake (% of control group)	100 ± 2^a^	60 ± 3^b^	53 ± 6^b^	62 ± 4^b^
Met equivalents consumed (g/day)	0.127 ± 0.003^a^	0.063 ± 0.003^b^	0.023 ± 0.003^c^	0.013 ± 0.001^d^
Met equivalents consumed (% of control group)	100 ± 2^a^	49 ± 1^b^	18 ± 2^c^	10 ± 2^d^

Values are means ± SEM for 4 rats. Values within a row not followed by the same superscript letter are significantly different at *P* ≤ 0.05 by ANOVA and Tukey's comparison.
